# Prevalence and factors associated with intergenerational sexual partnerships among undergraduate health science students in Eswatini

**DOI:** 10.4314/ahs.v22i2.50

**Published:** 2022-06

**Authors:** Faith Mamba, Mduduzi Colani Shongwe

**Affiliations:** 1 Southern Africa Nazarene University, Department of Nursing, Faculty of Health Sciences; 2 University of Eswatini, Department of Midwifery Science, Faculty of Health Sciences

**Keywords:** Age-mixing, cross-generational sexual partnerships, intergenerational sexual partnerships, tertiary students, Eswatini

## Abstract

**Background:**

Intergenerational sexual partnerships (IGSPs) put young people at an increased risk of sexually transmitted infections (STIs) including HIV and AIDS. However, their burden and predictors remain poorly understood.

**Objective:**

To describe the prevalence and factors associated with IGSPs among undergraduate health science students at a selected tertiary institution in Eswatini (formerly Swaziland).

**Methods:**

Data were collected through a cross-sectional survey of 148 conveniently sampled undergraduate health science students at the University of Eswatini. Multiple logistic regression analysis was performed to determine predictors of IGSPs (i.e. a 10-year or greater age disparity between heterosexual partners).

**Results:**

There was equal participation of males and females in this study (50%, n=74). The prevalence of IGSPs among the sample was 31.8% (n=47). Females (adjusted odds ratio, AOR = 0.08, 95% CI: 0.03–0.24) and those who received money from sexual partners (AOR = 0.08, 95% CI: 0.01–0.62) had lower odds of being in IGSPs.

**Conclusion:**

Being female and being a recipient of money from a sexual partner were negatively associated with IGSPs. The relatively high prevalene of IGSPs calls for health education campaigns targeting university students on the negative consequences associated with IGSPs, especially among young women.

## Introduction

Intergenerational sexual partnerships (IGSPs), also known as age-mixing or cross-generational relationships, are sexual relationships of people with an age difference of ten years or more^1^. IGSPs are not only uncommon in sub-Saharan Africa (SSA), including in Eswatini^2^, but also in other regions such as in North America^3^. Although such relationships occur mostly between young women and older men, a phenomenon known as “sugar daddies” or “blessers”^4^, they also occur between young men and older women, colloquially known as “sugar mamas or Ben 10s”^5^, ^6^. These phenomena have recently been trending in social local media platforms, especially among young social media users in Suthern African countries such as Zimbabwe^7^, South Africa^8^,^9^, Namibia^10^, Botswana^11^, and in Eswatini^2^. Eswatini, a small landlocked country in Southern Africa, with a population of about 1.1 million, of whom 51% are aged less than 24 years^12^. The country has one of the highest HIV prevalence in the world, at 27% among adults ≥ 15 years^13^, a sub-population that also includes tertiary students. In 2011, the Eswatini government slashed the amount of scholarship allowances for tertiary students by 60%, exposing them to dire financial conditions^14^; in a country where 20% of children (0–17 years) are double orphans, and 60% are classified as vulnerable^15^.

Studies have reported prevalences of IGPS in the general population of women and/or men e.g. 13.8% in Barbados^16^ and 9.5% in Nigeria^17^, and not specifically among tertiary students. However, one study conducted at a South African university, found that 50% of sexually experienced students had engaged in IGSPs^18^. In Eswatini, nationally representative surveys of the general population found prevalences of 14.1% among 15–24-year-old women in 2010^19^ and 22.5% among 20–24-year-old women in 2014^15^. A recent study^2^ to determine who were the male partners of adolescent girls and young women (15–24 years) in Eswatini revealed that 60% of the 843 men (aged 20 to 34 years) in the study had female partners aged 15–19 years, and 70% had partners aged 20–24, with 12% and 11% of these reporting five or more such partners in the previous 12 months, respectively.

Negative consequences of IGSPs include HIV and AIDS and sexually transmitted infections (STIs)^20^, unplanned pregnancies, sex work, and sexual violence^21^, risky sexual behaviours^22^, and emotional and physical victimization^23^. In Eswatini, unsafe sex practices, intergenerational sex, multiple concurrent partners, and misconceptions about HIV transmission account for the 9.1% HIV prevalence among young people aged 15–24 years^13^,^24^. Risk factors for IGSPs include poverty or poor socio-economic status^25^, having parents with lower education level, having friendships with older adolescents or peer pressure^26^, place of residence, food insufficiency, alcohol use^27^, feeling secure, being a student, marital status^8^, powerlessness, increased libido, curiosity, attitudes towards assertive women, culture and religion^28^, receiving money or gifts from a partner, age at sexual debut, self-esteem^16^, familial and developmental factors^3^.

Research on IGSPs is scarce compared to age-disparate research, and most of the studies on IGSPs are qualitative. Additionally, most of the studies included samples of either women or men^3^,^7^,^26^, and few have both^10^. Besides, in national surveys^15^,^19^, data collection is usually conducted within households during the day, yet a majority of tertiary students reside in university campuses, thus their views are likely to be under-represented in these studies. The problem is further compounded by that a majority of studies investigating IGSPs have been conducted under the assumption that IGSPs occur mainly between young unmarried women and older men, and less between older women dating young men^3^,^21^, yet that is not always the case^29^. In Eswatini, it is important to explore the “sugar mama” phenomenon among young boys as they have also been reported to be engaged in romantic relationships with older women^30^.

The Eswatini government and its partners have spearheaded numerous national initiatives aimed at reducing the incidence of HIV through behaviour change campaigns, such as the 2006 campaign dubbed “Makhwapheni (Your secret lover) can kill you”^31^, rolling out a compulsory HIV course to all freshmen in all tertiary institutions, introduction of life skills education in secondary and high schools and the “Ngitawutfolani... Ungayitfola neHIV” (What's in it for me... You can also get HIV) campaign, amongst others^32^. However, despite the above-mentioned initiatives, IGSPs are still prevalent^15^, raising a need for studies that will point out factors that put youth at risk of HIV to inform youth sexual health programming. Such sexual health programmes are spearheaded by healthcare workers, of which health science tertiary students are the future health workforce, thus the desired behavior change is expected to start with them. It is against this backdrop that we conducted this study to determine the prevalence and factors associated with IGSPs among undergraduate health science students at the University in Eswatini (UNESWA)'s Faculty of Health Sciences (FHS).

## Methods

### Study design and setting

This study employed a cross-sectional design and was conducted at UNESWA, FHS, located in Mbabane, the capital city of Eswatini. Currently, Eswatini has four major universities; three of which started operations after the year 2010. This university was purposively selected as it is the oldest and the largest university in Eswatini, with nine faculties, of which the FHS had four departments with a student enrollment of about 476 at the time of data collection.

### Study population

The study population comprised undergraduate students in levels III and IV at the UNESWA, FHS. To be part of this study, the students had to be older than 18 years and be pursuing an undergraduate health science degree, and be in Level III or IV. We excluded the Levels I and II students because we wanted to enroll participants who had had optimal exposure to the risk factors related to living a university life, and to ensure that any residual risk factors from high school life had been washed out.

### Sample size determination

In this study, the sample size was calculated using the online Raosoft sample size calculator^33^. Assuming a population size of 253 (based on student enrollment for the two-class levels in the year 2015/2016), a 95% confidence interval (CI), a margin of error of 5%, and a response distribution of 22.5% (based on the prevalence of IGSPs in the general population of women aged 20–24 years in Eswatini at the time)^15^, the minimum desired sample size for this study was 131. With a 10% contingency for non-response, we planned to recruit at least 145 participants. To increase the statistical power of the study and due to the fact such a source population size would be manageable in terms of research resources, initially, we decided to enroll all 253 of them. However, during the different days of data collection, a total of 201 students were present, 35 of whom refused to participate (citing busy schedules), while 18 questionnaires were not returned. As a result, 148 questionnaires were returned and retained for analysis, resulting in a 73.6% response rate.

### Measurements

A well-structured self-administered questionnaire adapted from a study by Drakes, Perks^16^ was used to collect the data. The questionnaire was pretested at the Southern Africa Nazarene University (SANU), FHS among 19 Level IV nursing students. The questionnaire was in English as it is the only instructional language at UNESWA. The primary outcome measure in this study was ever being in an IGSP i.e. a relationship with a 10-year or more age gap between heterosexual partners^1^. We adopted the UNAIDS definition because it does not confine IGSPs to unmarried young girls only, which makes it more relevant for a setting like Eswatini where both ‘sugar daddy’ and ‘sugar mama’ phenomena are not uncommon^30^,^34^. Being in an IGSP was ascertained using the question: “In your lifetime, have you ever had a sexual partner who is or was 10 or more years older than you (yes/no)?”

### Recruitment and data collection

We used convenience sampling to recruit the students, whereby all students who were present on the day of data collection were invited to participate. Through class representatives, students were asked to remain behind after their last class periods, and those who agreed to participate filled the questionnaire on the spot. On average, it took about 20–25 minutes to fill out the questionnaire. The data were collected in April 2016.

### Data analysis

Data were analyzed using IBM Statistical Package for Social Sciences (SPSS) version 20^35^. We performed Chi-square and Fisher's exact tests to compare socio-demographic characteristics of participants who had engaged in IGSPs with those who had not. The explanatory variables were identified during the literature review^8^,^16^,^25^–^27^,^39^,^43^. Multiple logistic regression analysis was performed using the augmented backward elimination strategy to select the covariates to be retained in the final model, hence we still retained non-statistically significant covariates due to their theoretical and clinical significance. Adjusted odds ratios (AORs) were computed alongside their 95% CIs at an alpha level of 0.05.

### Ethical considerations

Ethical clearance was granted by the then Scientific and Ethics Committee (SEC) of the Ministry of Health in Eswatini (ref: MH/599c/FWA00015267/IRB0009688). All ethical principles inherent in research involving humans were ensured according to the guidelines of the Declaration of Helsinki. Participation in the study was voluntary and written informed consent was obtained prior to par-ticipation. Privacy was ensured by asking participants to sit far apart during data collection and further not writing any identifying information on the questionnaires. The consent forms were not attached to the questionnaires to avoid linking responses to any of the participants. None of the university staff was present or involved during the data collection to ensure privacy and confidentiality.

## Results

### Participants' background characteristics

Of the 148 participants, 50% (n = 74) were female, 70.3% (n = 104) were aged 19–24 years, and 77% (n = 114) were single. More than half (54.1%, n = 80) did not have enough money to cover basic necessities and 73% (n = 108) had received money from sexual partners. About a third (31.8%, n = 47) reported having ever been in IGSPs. Chi-square tests showed that those who had ever been in IGSPs were statistically significantly different from those who had not by gender (p < 0.001), having money to cover basic necessities (p = 0.003), and having ever received money from sexual partners (p < 0.001) [Table 1].

**Table 1:** Background characteristics of participants by IGSP status (*N* = 148)

		Ever in IGSP^a^			
				
Characteristic	Total *n* (%)	Yes (*n* = 47) *n* (%)	No (*n* = 101) *n* (%)	χ^2^	*p*
**Age**				3.77	0.05
19–24 years	104 (70.3)	28 (59.6)	76 (75.2)		
≥25 years	44 (29.7)	19 (40.4)	25 (24.8)		
**Gender**				42.68	< 0.001
Female	74 (50.0)	42 (89.4)	32 (31.7)		
Male	74 (50.0)	5 (10.6)	69 (68.3)		
**Current marital status**				11.86	0.001
Single	114 (77.0)	28 (59.6)	86 (85.1)		
Married/Cohabiting	34 (23.0)	19 (40.4)	15 (14.9)		
**Religiosity** ^b^				0.37	0.54
Never/rarely	67 (45.3)	23 (48.9)	44 (43.6)		
Regularly	81 (54.7)	24 (51.1)	57 (56.4)		0.43
**Field of study**				0.64	
Environmental Health- related	41 (27.7)	11 (23.4)	30 (29.7)		
Nursing-related	107 (72.3)	36 (76.6)	71 (70.3)		
**Sponsor**				1.52	0.22
Government/Other organization	130 (87.8)	39 (83)	91 (90.1)		
Self-sponsored	18 (12.2)	8 (17.0)	10 (9.9)		
**Have enough money to** **cover basic necessities**				8.87	0.003
Yes	68 (45.9)	30 (63.8)	38 (37.6)		
No	80 (54.1)	17 (36.2)	63 (62.4)		
**Have enough money to** **cover luxuries**				0.40	0.53
Yes	23 (15.5)	6 (12.8)	17 (16.8)		
No	125 (84.5)	41 (87.2)	84 (83.2)		
**Important thing** **looking for in a** **relationship**				0.002	0.96
Money/Sex	28 (18.9)	9 (19.1)	19 (18.8)		
Love/Marriage	120 (81.1)	38 (80.9)	82 (81.2)		
**Ever received money** **from a sexual partner**^c^					< 0.001e
Yes	108 (73.0)	46 (97.9)	62 (61.4)		
No	40 (27.0)	1 (2.1)	39 (38.6)		

Notes: ^a^IGSP, intergenerational sex partnership; ^b^Religious services attendance per month; ^c^irrespective of their age; ^e^Fisher's exact test

### Factors associated with intergenerational sexual partnerships

After adjusting for other covariates in the model, female students (AOR = 0.08, 95% CI: 0.03–0.24, p < 0.001) vs male students, and those who had ever received money from sexual partners (AOR = 0.08, 95% CI: 0.01–0.62, p = 0.02) compared to those who had not, had lower odds of being in IGSPs (Table 2).

**Table 2:** Multivariate logistic regression model of factors associated with IGSPs (*N* = 148)

Variable	AOR (95 % CI)	*p*-value
**Age**		
19–24 years	2.70 (0.90, 8.16)	0.08
≥25 years	ref	
**Gender**		
Female	0.08 (0.03, 0.24)	< 0.001
Male	ref	
**Current marital status**		
Single	1.40 (0.47, 4.20)	0.55
Married/Cohabiting	ref	
**Have enough money to cover basic necessities**		
Yes	ref	
No	1.80 (0.72, 4.55)	0.21
**Ever received money from a sexual partner**		
Yes	0.08 (0.01, 0.62)	0.02
No	ref	

**Notes:** β for constant = 3.38; R^2^ = 0.37 (Cox & Snell), 0.52 (Nagelkerke); Model χ^2^ (5) = 68.02, p < 0.001; **Abbreviations:** IGSPs, intergenerational sex partnerships; COR, crude odds ratio; AOR, adjusted odds ratio; CI, confidence interval; ref, reference category

## Discussion

We found a 31.8% prevalence of IGSPs. Being female and having ever received money from a sexual partner were found to be associated with being in IGSPs. The prevalence of IGSPs in this study was higher than in local national surveys e.g. 14.1% among 15–24-year-old women in 2010^19^, 22.5% among 20–24-year-old women in 2014^15^, and in other studies e.g. 13.8% in Barbados^16^, but lower than the 50% reported among university students at the University of Kwa-Zulu Natal in South Africa^18^. Swati men (20–34 years) report an even higher prevalence of relationships with young girls (60% with girls aged 15–19 and 70% with girls ages 20–24 in the last 12 months)^2^. The possible reasons for the differences in the prevalence could be that our sample included only tertiary students, who might be a high-risk group for the “blesser-blessee” phenomenon due to their financial vulnerability during university life than the general population of young people used in other surveys^37^,^38^. The other reason for the differences in the prevalence lies in the measurement of IGSPs in the different studies i.e. lifetime versus previous 12 months such as in national surveys. Moreover, differences in study settings, age, gender composition, and years of data collection could also account for the differences in the prevalence in the different studies.

The negative association between being female and IGSPs was surprising as previous research^39^ has shown that females are more likely to engage in IGSPs. One possible reason for the negative direction could be the small number of males who reported to be in IGSPs in our sample (see Table 1). In bivariate analysis (Table 1), a higher proportion of females reported being engaged in IGSPs than males, in line with previous studies. Jones^40^ stated that from an early age, Swazi girls are enculturated with the view that their bodies are assets for transactions and thus place a high value on relationships with older men. Fielding-Miller, Dunkle^34^ also found that Swati women expected and received significant financial support from their male partners.

In this study, receiving money from a sexual partner was negatively associated with IGSPs, contrary to findings by Drakes, Perks^16^. This could be due to the small number of males who reported IGSPs and who reported receiving money from sexual partners in our sample (Table 1). Our sample included male participants, yet Drakes, Perks^16^ in-cluded only females from the general population, hampering direct comparisons between the two studies. In addition, in our study, financial gain was not the main motivation for being in romantic relationships, instead, the majority (80.9%) were looking for love or marriage in relationships (Table 1). Leclerc-Madlala^41^ observed that a lot of young girls think that older men are the marriageable type and more responsible, or because they are seeking affirmation of their value as women, or want to build social networks and capital, and/or want to boost their self-esteem and social status yet those men simply date them to fulfil their sexual motives^42^.

Similar to findings by Drakes, Perks^16^, in our study, not having enough money to cover basic necessities was not significantly associated with IGSPs, possibly due to that a majority of the students in our study were government-sponsored (Table 1), hence their allowances probably covered their basic needs. Likewise, in South Africa, De Wet, Alex-Ojei^8^ also found that financial motivations were not the predominant reason for age-disparate relationships among young women. In this study, marital status and age were not significantly associated with IGSPs, similar to findings by Weiser, Leiter^27^ among a sample of Swazi and Tswana women. However, they^27^ found a significant association among males. On the contrary, in Zimbabwe, Schaefer, Gregson^43^ found that marital status predicted age-disparate relationships among women (15–24 years). Unfortunately, a direct comparison of our findings with those from these studies e.g. Schaefer, Gregson^43^, is difficult since their study was on age-disparate relations and not IGSPs.

## Strengths and limitations of the study

Our sample included both males and females and is among the few that include married participants, which enabled us to explore the association between marital status and IGSPs. To our knowledge, this is the first study to be published on IGSPs among tertiary students in Eswatini, a high HIV prevalence setting. However, the study has some limitations. First, the small number of males who reported engaging in IGSPs limited our ability to conduct a gender-stratified analysis which could have teased out any gender differences in the predictors ofhe IGSPs. Second, since the questionnaire was self-reported, social desirability and recall bias cannot be completely ruled out in this study. Third, the cross-sectional nature of the study prevented us from determining temporality and causality since the independent variables and outcome of interest were measured simultaneously. Using the quantitative approach also limited our ability to get an in-depth understanding of the issues around IGSP and to explore their rationalities. Lastly, the study is also prone to selection bias due to the convenience sampling method employed when recruiting participants, thus limiting the generalizability of the findings to the studied setting.

## Conclusion and implications

The prevalence of IGSPs was 31.8% among the university students in the studied institution. Being female and having ever received money from a sexual partner were negatively associated with being in IGSPs. There is a need to reinforce health education messages on the negative consequences of IGSPs in order to curb this practice among tertiary students, especially among young women. UNESWA should capitalize on the recently opened community/campus radio station to reach out to students about key messages related to IGSPs. Health science lecturers should emphasize the need for undergraduate health science students to lead by example by practising safe sexual behaviors. There is a need to engage the students' leadership through the office of the Dean of Student Affairs to facilitate the start of income-generating clubs within the campus, and to encourage students to look for part-time employment such as in restaurants within the city to reduce their financial dependence on sexual partners. There is a need to replicate the study among students from other faculties within UNESWA and in all tertiary institutions in Eswatini. Finally, future qualitative studies on IGSPs are warranted to get an in-depth understanding of why tertiary students engage in IGSPs in Eswatini.
